# Targeted Degradation of 53BP1 Using Ubiquitin Variant Induced Proximity

**DOI:** 10.3390/biom12040479

**Published:** 2022-03-22

**Authors:** Bayonle Aminu, Julia Fux, Evan Mallette, Nathaniel Petersen, Wei Zhang

**Affiliations:** 1 Department of Molecular and Cellular Biology, College of Biological Science, University of Guelph, 50 Stone Rd E, Guelph, ON N1G 2W1, Canada; baminu@uoguelph.ca (B.A.); jfux@uoguelph.ca (J.F.); emallett@uoguelph.ca (E.M.); npeter06@uoguelph.ca (N.P.); 2 CIFAR Azrieli Global Scholars Program, Canadian Institute for Advanced Research, Toronto, ON M5G 1M1, Canada

**Keywords:** Ubiquitin Variant, targeted protein degradation, PROTAC, E3 ligase, 53BP1, NEDD4L, RFWD3

## Abstract

In recent years, researchers have leveraged the ubiquitin-proteasome system (UPS) to induce selective degradation of proteins by E3 ubiquitin ligases, which has great potential as novel therapeutics for human diseases, including cancer and neurodegenerative disorders. However, despite extensive efforts, only a handful of ~600 human E3 ligases were utilized, and numerous protein–protein interaction surfaces on E3 ligases were not explored. To tackle these problems, we leveraged a structure-based protein engineering technology to develop a multi-domain fusion protein bringing functional E3 ligases to the proximity of a target protein to trigger its proteasomal degradation, which we termed Ubiquitin Variant Induced Proximity (UbVIP). We first generated non-inhibitory synthetic UbV binders for a selected group of human E3 ligases. With these UbVs employed as E3 ligase engagers, we designed a library of UbVIPs targeting a DNA damage response protein 53BP1. We observed that two UbVIPs recruiting RFWD3 and NEDD4L could effectively induce proteasome degradation of 53BP1 in human cell lines. This provides a proof-of-principle that UbVs can act as a means of targeted degradation for nucleus-localized proteins. Our work demonstrated that UbV technology is suitable to develop protein-based molecules for targeted degradation and can help identify novel E3 ligases for future therapeutic development.

## 1. Introduction

Ubiquitination is a highly conserved intracellular protein post-translational modification pathway, which determines protein fate through conjugation of the small protein ubiquitin (Ub) to lysine residues of protein substrates [1,2]. Ubiquitination involves the action of three enzymes: the E1 activating enzymes, E2 conjugating enzymes, and E3 Ub ligases [3,4]. An important outcome of ubiquitination is directing ubiquitinated protein substrates to the 26S proteasome for degradation [2]. Hence, misfolded, damaged, and short-lived regulatory proteins involved in various biological processes are naturally targeted for degradation by the ubiquitin-proteasome system (UPS) [5]. In recent years, many research and industrial development efforts have aimed at leveraging the UPS to selectively and precisely degrade proteins of interest to study their biological functions and to exploit the therapeutic potential of this method [6]. One of the most studied targeted protein degradation strategies is proteolysis targeting chimeras (PROTACs, also known as bifunctional degraders), which use two distinct binding moieties: one that binds to a target protein and the other that recruits the E3 Ub ligase with a linker connecting the two [7,8,9].

PROTACs allow simultaneous binding of a E3 ligase and a target protein (separate from the native E3 ligase substrate), thereby forming a ternary complex. Due to the proximity of the E3 ligase, ubiquitination of the target will be catalyzed and this leads to subsequent proteasomal degradation [10]. So far, more than 40 cellular targets have been successfully degraded by PROTACs [11] using chemical compounds. However, a couple of limitations exist for PROTACs. Firstly, only four out of over 600 E3 ligases encoded in the human genome have been thoroughly studied and repeatedly utilized. The vast majority of PROTACs that have been described in the literature over the last few years are based on a selective binder of two E3 ligases: von Hippel–Lindau (VHL) and cereblon (CRBN) [12]. Secondly, obtaining small molecule binders in the absence of well-defined binding sites with flat and shallow protein interface for certain proteins of interest is extremely difficult. These restrictions have greatly impaired PROTACs development and put significant restraint on the range of proteins a PROTACs can target. To tackle these problems, rapid development of protein or peptide binders for targeted protein degradation is in urgent need [13].

Over the past five years, a structure-based combinatorial protein engineering strategy has been used to systematically develop Ubiquitin Variants (UbVs) as modulators of human E3 ligases across different sub-families [14]. UbV technology involves subjecting the wild type Ub scaffold to rational protein design, where mutations are introduced on the binding surface of Ub with other UPS proteins to produce a phage-displayed UbV library and followed by selections of UbVs that bind to the target substrate with high affinity and specificity [15]. In general, UbVs have been shown to target protein–protein interactions in a potent and specific manner, which gives an advantage over small molecule binders. This strategy has been used to obtain binders that act as inhibitors for E3 ligases [16,17,18], due to hijacking the E2~Ub binding sites with high-affinity binding. Importantly, UbV activators were developed for HECT E3 ligases [16]. The activators were found to increase autoubiquitination, formation of polyubiquitin chains and substrate ubiquitination in NEDD4L and WWP1 by binding to a N-lobe exosite on the E3 ligases [16]. UbV activators have also been identified for a homodimeric RING E3 ligase XIAP, stabilizing its dimerization thereby causing an increased catalytic activity in vitro and in cells [17]. The ability of UbVs to serve as activators of E3 ligases in different classes indicates their potential in recruiting E3 ligases for targeted degradation.

Here, we presented a proof-of-concept study of Ubiquitin Variant Induced Proximity (UbVIP). UbVIPs function similarly to PROTACs by recruiting an E3 ligase to a target protein, therefore mediating target degradation. It is distinct from PROTACs because the components are all peptide based rather than small molecules, and UbVIPs use UbVs to specifically recruit novel E3 ligases in targeted degradation. In this study, we first conducted phage-display selections and identified high affinity non-inhibitory UbV binders for several human E3 ligases including RFWD3, UBE3A, and MDM2. In addition to these binders, we included a previously published UbV activator of NEDD4L [16] as E3 ligase “engagers”. Afterwards, we designed a UbVIP library to link these E3-binding UbVs to a 53BP1-binding UbV [16] to screen for UbVIPs that can induce targeted degradation of 53BP1. Two UbVIPs were identified as effective in degrading 53BP1 in a proteasome-dependent manner. We also showed that the UbVIPs bind to their respective E3 ligases and 53BP1 simultaneously to form a potential ternary complex. This work sheds light on the application of novel E3 ligases in targeted protein degradation and provided synthetic molecules for the research community to design and target various nucleus-localized proteins for degradation.

## 2. Materials and Methods

### 2.1. Phage Display and Ubiquitin Variant (UbV) Selections

The following proteins were subjected to UbV selections: RFWD3^WD40^ (provided by Dr. Yufeng Tong), UBE3A^HECT^ (provided by Dr. Sachdev Sidhu), and MDM2^RING^ (provided by Dr. Danny Huang). The phage-displayed UbV library used for selection was re-amplified from Library 2 as previously reported [15]. Protein immobilization and the following phage selections were done according to standard protocols [16]. Briefly, purified E3 ligases were coated on 96-well MaxiSorp plates (Thermo Scientific, Mississauga, ON, Canada 12565135) by adding 100 µL of 1 µM proteins and incubating overnight at 4 °C. Five rounds of phage display selections were performed as follows: (a) preparation of the phage library pool, within which each phage particle displays a unique UbV and encapsulates the UbV encoding DNA in a phagemid; (b) the pool of phage-displayed UbV library are applied to immobilized protein targets; (c) E3 ligase-binding phages are captured, and non-binding phages are washed away; (d) bound phages are amplified by infection of bacterial host *E. coli* (OmniMAX); (e) individual phages with improved binding properties obtained from round 4 and round 5 are identified by phage ELISA (see below) and subjected to DNA sequencing of the phagemids to obtain UbV sequences.

### 2.2. ELISA Binding Assays

Proteins in study were immobilized on 384-well MaxiSorp plates (Thermo Scientific, Mississauga, ON, Canada 12665347) by adding 30 µL of 1 µM proteins for overnight incubation at 4 °C. Phage and protein ELISA against immobilized proteins was performed as described [16]. Binding of phages was detected using anti-M13-HRP antibody (GE Healthcare, Mississauga, ON, Canada 27942101) and binding of FLAG-tagged UbVs was detected using anti-FLAG-HRP antibody (Sigma-Aldrich, Oakville, Canada A8592). To measure the half maximal binding concentration (EC_50_) of UbVs binding to E3 ligases, the concentration of UbVs or wild type Ub was varied from 0 to 4 µM (24 points, 1:2 dilution), while the concentration of target proteins immobilized on the plate remained at 1 µM. EC_50_ values were calculated using the GraphPad Prism software with the built-in equation formula (non-linear regression curve).

### 2.3. UbV Expression and Purification

PCR amplified DNA fragments encoding the indicated UbVs with a N-terminal FLAG epitope tag were cloned into Gateway Entry vector pDONR221 (Thermo Scientific) according to the manufacturer’s instructions and then transferred into Gateway Destination expression vector pET53 (His-tagged, Thermo Scientific). The above-mentioned plasmids were used to transform Escherichia coli BL21 (DE3) for protein expression. Protein expression was induced by addition of IPTG (isopropyl β-D1-thiogalactopyranoside, Bioshop) at mid-log phase to a final concentration of 1 mM. After incubation overnight at 16 °C with shaking, cell pellets were collected by centrifugation (12,200× *g*, 10 min) and lysed, and proteins were purified using Ni-NTA Agarose (Qiagen, Toronto, ON, Canada 30250) at 4 °C following the manufacturer’s instructions. The purity of eluted fractions was determined by polyacrylamide gel electrophoresis. Protein concentrations were determined by measuring the absorption at 280 nm (Nanodrop 1000, Thermo Scientific). Eluted proteins were dialyzed into 50 mM HEPES buffer pH 7.5, 250 mM NaCl, 5% glycerol, and 1 mM DTT and stored at 4 °C or frozen at −80 °C for further applications.

### 2.4. In Vitro Ubiquitination Assays

Biochemical reactions to study E3 ligase autoubiquitination activity were performed in a volume of 25 µL in a buffer of 50 mM Tris pH 8.0, containing E1/UBE1 (50 nM, Boston Biochem, Cambridge, MA, USA), E2 as indicated (Boston Biochem, Cambridge, MA, USA), ubiquitin (20 µM, Boston Biochem, Cambridge, MA, USA), E3 ligases as indicated (1 µM), and UbVs (10 µM). After incubation at room temperature for 60 min, reactions were stopped by the addition of 10 mM EDTA and SDS-PAGE sample buffer and resolved using 4–20% gradient gel (Bio-Rad, Mississauga, ON, Canada 4561096). Poly-ubiquitinated E3 ligases were evaluated by western blotting.

To study p53 ubiquitination by MDM2 or UBE3A, a p53 Ubiquitination Kit (K-240, Boston Biochem, Cambridge, MA, USA) was used. Reaction buffer, p53, E1 enzyme, E2 enzyme, E6AP/UBE3A, Ub, and ATP were provided in the kit. We used the provided UBE3A to conduct the UBE3A-mediated p53 ubiquitination assay and replaced the E3 ligase to MDM2 for MDM2-mediated p53 ubiquitination assay. After mixing the reagents and incubation at 37 °C for 60 min, reactions were stopped by the addition of 2 μL 1M DTT and SDS-PAGE sample buffer and resolved using 4–20% gradient gel (Bio-Rad 4561096). Poly-ubiquitinated p53 were probed by Western blotting using anti-p53 antibody provided in the kit.

### 2.5. Cell Lines and Culture Conditions

U2OS cells were a kind gift from Durocher lab (University of Toronto), HEK293 and HeLa cells were generously donated by Uniacke lab (University of Guelph). The cells were kept at the stable atmosphere of 5% CO_2_ and 37 °C. U2OS cells were grown in McCoy’s medium supplemented with 10% fetal bovine serum (Corning), while HEK293 and HeLa cells were propagated in high glucose Dulbecco’s Modified Eagle Medium (DMEM) supplemented with 10% fetal bovine serum (Corning) and 1 mM Sodium pyruvate (Invitrogen). A volume of 1 mM Antibiotic-antimycotic (Gibco, Waltham, MA, USA 15240062) was added to all media. Cells were regularly tested for the presence of mycobacteria. All experiments were conducted with cells between passages 7–35.

### 2.6. Plasmids and Transfection

All UbVIPs with sequences coding for an N-terminal FLAG-tag were cloned on pcDNA3.1 plasmid after codon optimization for mammalian expression, all carried out by BioBasic. DNA sequences are available upon request. Once received, the plasmids were amplified in DH5α bacterial strain and purified with Qiagen maxi-prep kit. Cultured cells were grown in 6 well plates (Nunclon) and transfected using Lipofectamine 3000 reagent (Invitrogen), following manufacturers protocol, and the cells were collected 24 h post-transfection.

### 2.7. Protein Detection Using Western Blot and Chemiluminescence

Cells were lysed using NP40 cell lysis buffer (Invitrogen, Burlington, Canada FNN0021), supplemented with Halt protease inhibitor cocktail (Thermo-Scientific PI87786). The cell lysates were diluted with PBS to 25 mg/mL, and concentration was measured using DeNovix spectrophotometer. In general, 50–100 μg of protein samples was loaded for sodium dodecyl sulfate-polyacrylamide gel electrophoresis (SDS-PAGE) using a 4–15% gradient gel (Bio-Rad 4561086) and transferred onto 0.2 μm PVDF membranes (Cytiva, Toronto, ON, Canada 10600021). The membranes were blotted by the indicated antibodies: anti-FLAG-HRP (Millipore Sigma, Oakville, ON, Canada A8591), anti-53BP1 (Novus Biologicals, Toronto, ON, Canada NB100-304), anti-RFWD3 (Abcam ab138030), anti-NEDD4L (Proteintech, Rosemont, USA 10091-010), anti-RhoB (Santa Cruz, Dallas, TX, USA sc-108), anti-RPA32 (Bethyl Laboratories, Montgomery, USA A300-244A), anti-Ub (clone FK2, Millipore 04-263), and anti-β-actin (Thermo Scientific, Mississauga, ON, Canada MA515739). SuperSignal West ECL (Thermo Scientific, Mississauga, ON, Canada PI34579) was used to image the bands with Bio-Rad ChemiDoc XRS+ Imaging system. 

### 2.8. Detection of Intracellular Protein Interaction Using Co-Immunoprecipitation

Cells were grown on 150 mm dishes and treated as described above to obtain a sufficient amount of whole cell lysate (lysate/elute ratio = 1:100). Dynabeads tagged with G protein (Thermo Scientific, Mississauga, ON, Canada 10003D) were used in combination with M2 anti FLAG antibody (Sigma-Aldrich, Oakville, Canada F1804-200UG) to pull down the UbVIPs. The Dynabeads were first incubated with the antibody for 10 min at RT and then with the whole cell lysate for 1 h at 4 °C. The target proteins were eluted from the beads with 50 mM glycine pH 2.8 and neutralized with 1M Tris pH 7.5. Western blotting was then performed to show the bands that co-precipitated with the UbVIPs.

### 2.9. Epi-Fluorescent Microscopy

Cells were seeded into the Nunclon 8-well chamber slides (Thermo Scientific, Mississauga, ON, Canada 1154534) and allowed to adhere overnight. The cells were then transfected with either an empty vector or one of the UbVIPs. Then, 24 h post-transfection, the cells were fixed with 2% paraformaldehyde; treated with 0.3% Triton-X100; and then blocked with a buffer containing 10% newborn calf serum, 0.5% Igepal C-630, and 0.5% Saponin dissolved in PBS. The slides were then stained with antibody mix to visualize both UbVIP and 53BP1. Mounting medium with DAPI (Invitrogen™ ProLong™ Gold P36935) was used. The slides were imaged on EVOS M5000 microscope using 405, 488, and 594 nm lasers.

For image analysis, 6 images were taken for every well. All cells in every image were measured for 53BP1 levels unless the nucleus appeared fractured or multiple bright foci were present. The cells were then separated based on UbVIP expression (detected using the red channel). For the MG132 experiment, HeLa cells were treated for 4 h with either 5 µM MG132 or DMSO following transfection with UbVIP and 24 h incubation.

### 2.10. MTT Survival Assay

Cells were seeded in 48 well plates and allowed to adhere overnight and then transfected with either UbVIP plasmid or an empty vector. After the completion of the incubation period, 3-(4,5-dimethylthiazol-2-yl)-2,5-diphenyltetrazolium bromide (Tocris, Toronto, ON, Canada 5224) dissolved in PBS was added to the wells at 0.5 mg/mL final concentration. Following 1 h incubation at 37 °C, the media in the wells was replaced with 0.2 mL dimethyl sulfoxide per well. The plate was placed on a shaker for 30 min, and then, the plate reader (Biotek Synergy LX, Mississauga, ON, Canada) was employed to read the absorbance at 492 nm. The plate reader was set to take 25 readings of each well. For each sample, there were 3 wells on a plate. The experiment was repeated 3 times.

### 2.11. Statistical Analysis

All experiments were performed in triplicates. Celleste Image Analysis software and Graph Pad Prism software were used to interpret the results of immunohistological experiments. Unpaired *t*-test with Welch correction analysis was used, and *p*-value of 0.005 was defined as statistically significant.

## 3. Results

### 3.1. Identification of Non-Inhibitory UbV Binders for Selected E3 Ligases

**Figure 1:**
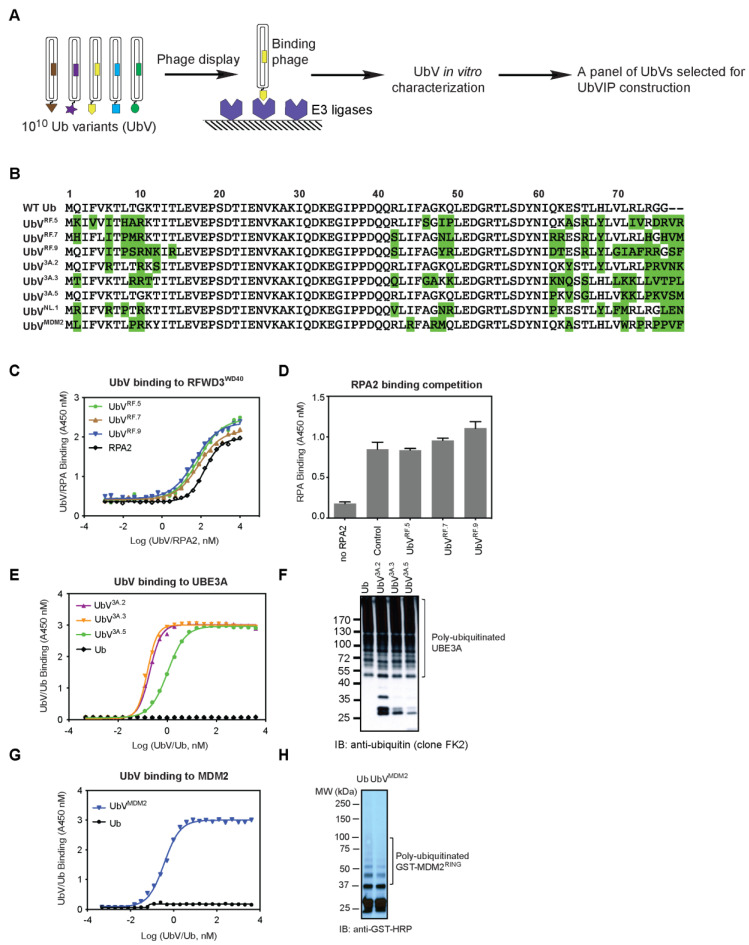
Development of non-inhibitory UbV binders for human E3 ligases. (**A**) Schematic representation of the UbV nomination process. Within the UbV library, each phage particle displays a unique UbV. Binding phages were captured with immobilized E3 ligases. After a total of five rounds of phage display selection, individual binding clones were subjected to sequencing. UbV binders were then characterized for their effects on known E3 ligase biological functions, and a total of six non-inhibitory UbVs were selected for UbVIP construction. (**B**) Protein sequences of the non-inhibitory UbVs (UbV^RF.5^, UbV^RF.7^, UbV^RF.9^, UbV^3A.2^, UbV^3A.3^, UbV^3A.5^, UbV^NL.1^, and UbV^MDM2^). Only the substitutions across the randomization surface of wild type Ub (WT Ub) are shown. It should be noted that UbVs have two amino acid extensions at the C-terminal (position 77 and 78). Dashes indicate conservation of the WT Ub sequence. (**C**) Binding curves of RFWD3 UbVs and RPA2 to RFWD3^WD40^, measured by ELISA (*n* = 3). The half maximal binding concentrations (EC_50_) of UbVs and RPA2 to RFWD3^WD40^ were determined by established methods [15] and are listed in the text. RFWD3^WD40^ (1 µM) were immobilized in microtiter plates. Serial dilutions of FLAG-tagged UbV or GST-tagged RPA2 (0–4 µM) were added and incubated for 20 min at room temperature. Wells were washed, and bound UbV/RPA2 was detected by anti-FLAG/GST-HRP conjugate antibody and colorimetric development of TMB peroxidase substrate. The absorbance at 450 nm (*y*-axis) was plotted against Log (UbV/RPA2 concentration, nM) (*x*-axis). (**D**) RPA2 binding to RFWD3^WD40^ was not affected by UbV^RF.5^, UbV^RF.7^, or UbV^RF.9^. Competition ELISA assays were performed as described in (**C**) for binding between RFWD3^WD40^ (immobilized and pre-incubated with UbVs or RPA2. (**E**) Binding curves of UBE3A UbVs to UBE3A^HECT^, measured by ELISA (*n* = 3). Experiments were conducted as described in (**C**). (**F**) UBE3A^HECT^ protein (pre-mixed for 15 min with WT Ub or UbV as indicated) was incubated for 1 h at room temperature with E1 (UBE1), E2 (UBE2L3), ATP, and Ub. Western blots were probed with an anti-Ub antibody (clone FK2) to detect mono- and poly-ubiquitinated UBE3A^HECT^. UbVs are not incorporated into chains because their C termini do not contain a di-glycine motif that is required for recognition by the E1 enzyme. (**G**) Binding curves of UbV^MDM2^ to MDM2^RING^, measured by ELISA (*n* = 3). Experiments were conducted as described in (**C**). (**H**) As in (**F**), autoubiquitination of MDM2^RING^ was assessed with control (WT Ub) or UbV^MDM2^.

We hypothesized that a successful UbVIP construct needs three parts: (1) a E3-binding module, (2) a target-binding module, and (3) a linker between the two modules. Therefore, we first set out to generate UbVs that are non-inhibitory for E3 ligases, yet show higher binding affinity than wild type Ub (WT.Ub). To accomplish this, we mined previous and ongoing UbV selections in our laboratory and identified eight non-inhibitory UbV binders for human E3 ligases (Figure 1A,B). Six of these UbVs were then subjected to UbVIP construction later in this work.

The first E3 ligase of interest we targeted is RFWD3 (RING finger and WD repeat domain 3). RFWD3 contains a N-term RING catalytic domain, and its C-term contains a WD40 domain to engage in protein–protein interactions with substrates [19]. RFWD3 ubiquitinates multiple sites on the RPA (replication protein A) heterotrimeric complex that binds single-stranded DNA generated during replication fork stalling and is necessary for recovery and homologous recombination at stalled forks [20]. Because WD40 domains have previously been shown to bind Ub [21], we set out to select for high-affinity binding UbVs of RFWD3^WD40^. Five rounds of phage display selections were conducted, and we obtained ten UbV binders for RFWD3^WD40^. Among them, three UbVs (UbV^RF.5^, UbV^RF.7^, UbV^RF.9^, sequences shown in Figure 1B) were found to bind to RFWD3^WD40^ better than RPA2 (Figure 1C, EC_50_ = 75 nM, 77 nM, and 48 nM, respectively; EC_50_ for RPA2 is 152 nM). Importantly, competitive ELISA showed that none of the three UbVs prevent RPA binding to RFWD3^WD40^ (Figure 1D), suggesting that native biological activity of RFWD3 is not affected.

The second E3 ligase of interest is a HECT E3 ligase UBE3A (also known as E6AP) [22]. As described above, we performed phage display selections and obtained 5 UbV binders for UBE3A^HECT^. Among them, three UbVs (UbV^3A.2^, UbV^3A.3^, and UbV^3A.5^; sequences shown in Figure 1B) bind to UBE3A^HECT^ with high affinity (Figure 1E, EC_50_ = 0.2 nM, 0.2 nM, and 1 nM, respectively). Notably, no binding was detected for WT Ub even at the highest concentration assayed (4 μM). We then monitored UBE3A autoubiquitination upon incubation with control or UbVs, and none of the UbVs affected intrinsic E3 ligase enzyme activity for UBE3A (Figure 1F). We further confirmed that the UbVs are non-inhibitory as they did not compromise UBE3A in ubiquitinating its substrate p53 (Figure S1). It should be noted that we have systematically developed inhibitors and activators for 20 human HECT-family E3 ligases but were not able to generate UbV binders for UBE3A at that time [16]. Nevertheless, we also included a previously published NEDD4L UbV activator (UbV^NL.1^) for the UbVIP design (Figure 1B).

Last but not the least, MDM2 is a RING E3 ligase with high protein expression level and activity in all cell types [23,24]. The RING domain of MDM2 confers its E3 ligase activity and is necessary for dimerization with itself or with the MDMX RING domain [25]. We performed UbV selections targeting the MDM2^RING(419-C)^ homodimer, and only one unique binder was found: UbV^MDM2^. While WT Ub did not bind MDM2 at the highest concentration assayed (4 μM), UbV^MDM2^ bound to MDM2 with high affinity (EC_50_ = 0.4 nM) (Figure 1G). Further characterization of UbV^MDM2^ showed that it is not an inhibitor of MDM2 based on effects on the MDM2 auto-ubiquitination assay (Figure 1H) and p53 ubiquitination assay (Figure S1).

### 3.2. Design and Cellular Expression Analysis of a UbVIP Library

To test the possibility of UbVIP-mediated proteasomal degradation of an intracellular protein target, we generated a library with each UbVIP molecule containing an E3-binding UbV, target-binding UbV, and a linker. The E3-binding UbVs included newly generated UbV^RF.5^, UbV^RF.7^, UbV^RF.9^, UbV^3A.2^, UbV^MDM2^ as described above, and a previously published UbV^NL.1^ [16]. The linkers selected for the UbVIP design are 8aa or 15aa Glycine (Gly, G)-Serine (Ser, S) linker (GGGGSGGG & GGGGSGGGGSGGGGS, respectively). It is important to consider the linker design because the length and flexibility of the linker can affect the activity of a degrader molecule [26]. Gly is commonly found in flexible natural linkers, and the addition of a polar Ser residue maintains stability of the linker [27]. Hence, the most used flexible linkers contain Gly and Ser residues, an example of which is the (Gly-Gly-Gly-Gly-Ser)_n_ sequence [27]. The two lengths used in our design were selected to represent medium and large linker lengths [28] to optimize adequate separation of the UbVIP components while maintaining necessary interactions. The target-binding module we selected is i53, a previously generated UbV that binds to 53BP1 selectively [29]. When tested in human and mouse cells, i53 was found to block accumulation of 53BP1 at DNA damage sites as observed by ionizing radiation focus formation, without reducing global 53BP1 protein levels [29]. i53 binds to the Tudor domain of 53BP1 with higher affinity than the native substrate: dimethylated histone H4 Lys20. In addition, when i53 was tested using ELISA assays and IP-MS experiments, it was found to bind specifically to 53BP1 [29]. Therefore, i53 specifically targets 53BP1 to prevent its recruitment to DNA damage sites and acts as inhibitor of 53BP1 function. To confirm the activity of i53, we transfected i53 into several cell lines alongside an empty vector control. The presence of 53BP1 at the spontaneous DNA damage sites manifests as bright dots (foci) that are visible in both U2OS and HeLa cells (Figure S2). However, the i53 transfected cells show an almost complete reduction in the number of foci, with 53BP1 only present in a non-aggregated form (Figure S2). In the end, the UbVIP library was a permutation of 6 different E3-UbVs, 2 different linkers, and 1 target-binding UbV and was arranged in two different orientations from N-term to C-term (UbV.E3-linker-i53 or i53-linker-UbV.E3) to form 24 UbVIP constructs (Table 1).

**Figure 2:**
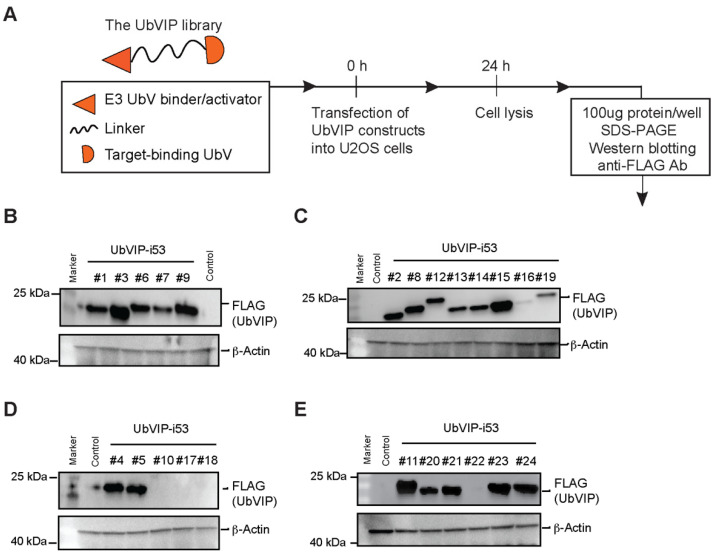
Cellular expression analysis of the Ubiquitin Variant Induced Proximity (UbVIP) library targeting 53BP1. (**A**) Pipeline for determining expression of UbVIP in mammalian cells. U2OS cells were transfected with the UbVIP constructs, and cells lysed after 24 h and whole cell lysates were subjected to SDS-PAGE and immunoblotting using an antibody against the FLAG tag. (**B**–**E**) Western blot analysis of UbVIP expressed in U2OS cells compared to untransfected control. The identifier numbers are as described in Table 1.

We determined the amino acid sequences of the 24 UbVIP constructs and then cloned into pcDNA3.1(+) with a FLAG tag (DYKDDDDK) at the N-term with DNA codon optimization for mammalian cell expression. To evaluate the expression of the UbVIPs in mammalian cells, we transfected U2OS cells and incubated for 24 h before whole cell lysates were probed for FLAG-UbVIP expression using Western blotting (Figure 2A). We observed that the UbVIP-i53 #3, #9, #11, #15 had the highest level of expression (Figure 2B–E). Interestingly, the E3-binding UbV in three of these four UbVIPs is UbV^RF.9^, showing that this particular UbV is expressed well even as part of a multi-domain protein. In contrast, there was no expression of UbVIP-i53 #10 and #22 that had UbV^3A.2^ as the E3 engager, which could also be an indication that some UbVs might not be expressed as expected when present in a multi-domain protein. We noted that UbVIP-i53 #16, #17, #18, and #19 showed the lowest level of expression among all, yet their corresponding reverse-orientation UbVIPs (#4, #5, #6, and #7, respectively) have normal expression level (Figure 2B–E). This suggested that orientation plays a role in UbVIP cellular expression, and the E3-binding module is preferably placed at the N-terminus.

### 3.3. Reduction of 53BP1 Protein Level in the Presence of Selected UbVIPs

**Figure 3:**
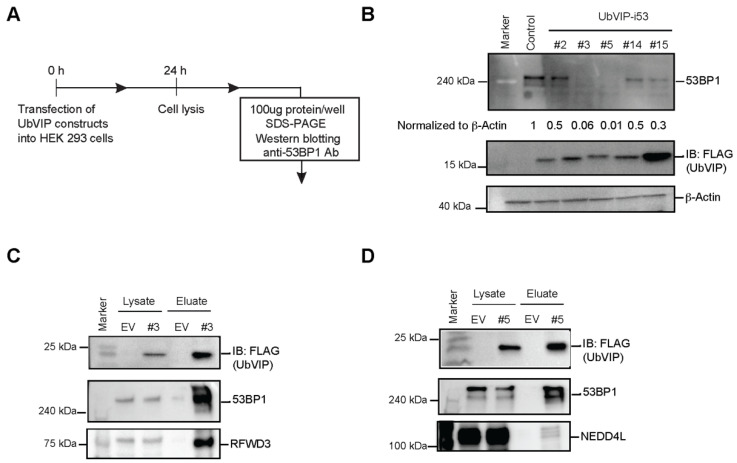
Two UbVIPs recruited cellular E3 ligases to induce 53BP1 degradation. (**A**) Experimental outline for determining 53BP1 protein expression in mammalian cells. HEK 293 cells were transfected with the UbVIP constructs and cells lysed after 24 h and whole cell lysates were subjected to SDS-PAGE and immunoblotting using an anti-53BP1 antibody. (**B**) Western blot analysis of 53BP1 expressed in HEK 293 cells compared to untransfected control, presence of UbVIP was confirmed by blotting for FLAG. β-Actin was used as a loading control. The UbVIP-i53#3 and #5 corresponding to UbV^RF.9^-8aa-i53 and UbV^NL.1^-8aa-i53 respectively, caused a reduction in 53BP1 levels when compared to UbVIP with the same targets but different orientation. 53BP1 protein abundance was measured using ImageJ software and normalized to β-Actin. Values indicated are relative to the untransfected control. (**C**,**D**) UbVIP-i53#3 and #5 interact with 53BP1 and corresponding E3 ligases (RFWD3 and NEDD4L). Immunoprecipitation (IP) of Flag-tagged proteins from extracts prepared from HeLa cells transfected with vectors expressing UbVIP-i53 #3 and UbVIP-i53 #5 or the empty vector control. Proteins were separated by SDS-PAGE and immunoblotted (IB) for FLAG, 53BP1, RFWD3 in (**C**) and NEDD4L in (**D**).

To determine whether the intracellular expression of UbVIP leads to 53BP1 degradation, we probed for endogenous 53BP1 of the whole cell lysates collected from cells transfected with UbVIP for 24 h (Figure 3A). Through Western blotting, we observed the expression of endogenous 53BP1 in HeLa, HEK293, and U2OS cells and compared with the changes in protein levels after transfection with each of the 24 UbVIP plasmids. We found that in cells transfected with UbVIP-i53#3 (UbV^RF.9^-8aa-i53, RFWD3 as the E3 ligase) and UbVIP-i53#5 (UbV^NL.1^-8aa-i53, NEDD4L as the E3 ligase), there was a significant decrease (>90% based on quantitation) in 53BP1 protein abundance (Figure 3B). It was interesting to observe that UbVIP-i53#15 (i53-8aa-UbV^RF.9^, with a reverse orientation to UbVIP-i53#3) showed a weaker yet substantial reduction (70%) in 53BP1 protein level (Figure 3B). This suggested that the presence of E3 engager at the N terminus is probably favorable to the effectiveness of the UbVIP molecule.

We then probed native substrates of RFWD3 and NEDD4L as well as global ubiquitination levels to see whether the expression of UbVIP-i53#3 or #5 led to unwanted side effects. We found that UbVIP-i53#3 expression did not affect abundance of RFWD3 substrate RPA32 [20] or cellular ubiquitinated proteins (Figure S3A). Similarly, UbVIP-i53#5 expression did not affect abundance of NEDD4L substrate RhoB [16] or cellular ubiquitinated proteins (Figure S3B). We also conducted MTT assay that measures cellular metabolic activity as an indicator of cell viability, proliferation, and cytotoxicity. We noted a mild reduction of cell survival 24 h post transfection of UbVIP-i53#3 or #5 in both HEK293 and HeLa cell lines (Figure S3C). 53BP1^−/−^ MEF cells has prolonged accumulation in G2/M and increased chromosomal instability [30]. This may contribute to the decreased cell survival upon expression of UbVIP-i53 #3 and #5, which induce 53BP1 degradation.

Since we identified UbVIP-i53#5 that contains a previously published UbV (UbV^NL.1^) as an effective 53BP1 degrader, we then designed three additional UbVIPs (Table S1) that contained previously published E3 ligase UbV activators, namely, UbV^P2.1^ and UbV^P2.3^ [16] that activate WWP2, and UbV^XR^ that activates XIAP [17]. We transfected them into HEK293 cells and measured 53BP1 protein levels. We found that there was a reduction of 53BP1 protein level when compared to the control but much weaker than UbVIP-i53#3 and #5 (Figure S4). This indicates that in addition to the linker length and orientation, the specific E3 engager also plays an important role in the design of an effective UbVIP molecule.

Next, we wanted to confirm that the observed 53BP1 degradation upon expression of UbVIP-i53#3 and #5 is due to the interaction of the UbVIPs with E3 ligases and 53BP1. We performed a co-immunoprecipitation where UbVIPs were pulled down from the whole cell extract using magnetic beads followed by Western blotting to confirm the presence of both 53BP1 and the E3 ligase, in the eluate. The pull-down results show that the constructs UbVIP-i53#3 and UbVIP-i53#5 bind the target E3 ligases—RFWD3 and NEDD4L, respectively—while simultaneously interacting with cellular endogenous 53BP1 (Figure 3C,D). These results, in combination with the 53BP1 protein abundance data, provided strong evidence that UbVIPs recruit E3 ligases to initiate the 53BP1 degradation cascade.

### 3.4. UbVIP-i53#3 and #5 Target 53BP1 for Proteasome-Mediated Degradation

**Figure 4:**
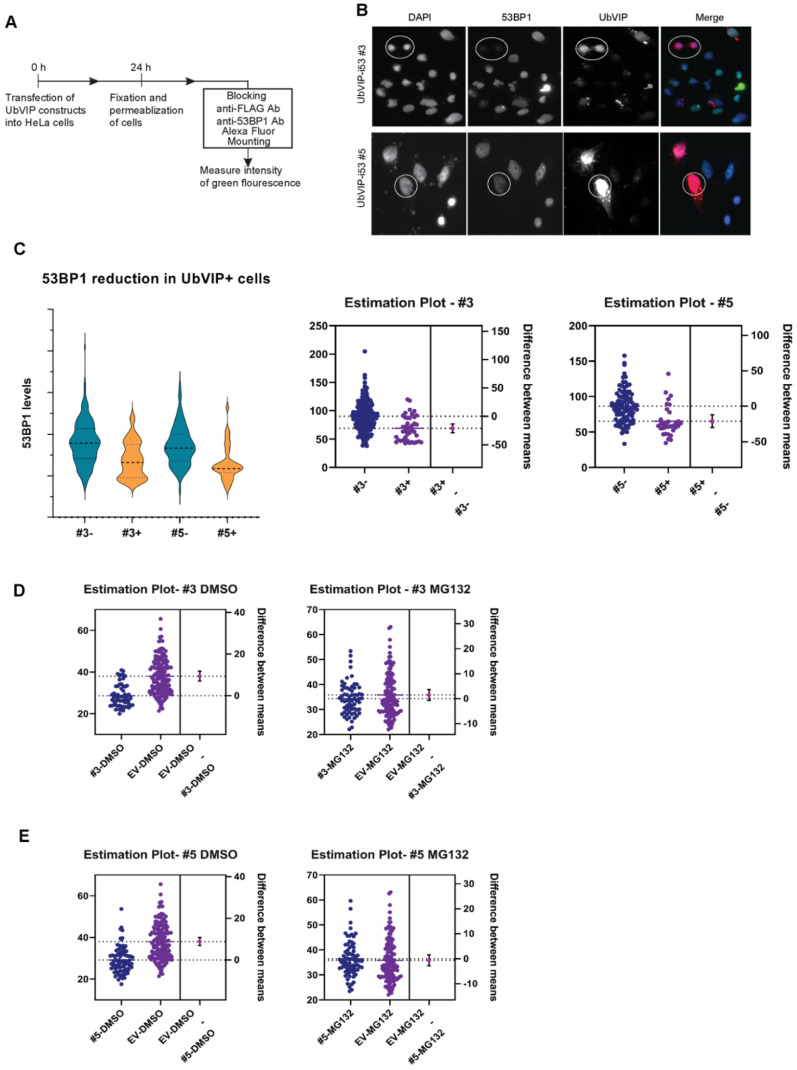
(**A**) Experimental outline for determining 53BP1 presence using immunofluorescent analysis of HeLa cells. HeLa cells were transfected with the UbVIP constructs and after 24 h, cells were fixed and stained with antibody against 53BP1 and FLAG tag. Cells were observed microscopically and intensity of 53BP1 fluorescence was measured. (**B**) Representative micrographs of cells transfected with vectors expressing UbVIP-i53#3 and #5 and processed for immunofluorescence with the indicated antibodies 24 h post transfection. DAPI staining was used to delineate the cell nuclei and FLAG shows the presence of UbVIP. The cells in the circled region show cells that had the UbVIP present and the intensity of 53BP1 is compared to cells that do not have the UbVIP. (**C**) Quantitation of the experiment where UbVIP expressing cells (#3+, #5+) were compared to the ones without discernible expression of UbVIP (#3−, #5−) (*n* = 3). Total cell numbers for #3−, #3+, #5−, and #5+ are 357, 205, 187, and 149, respectively. All the cells in each image were counted to avoid bias and violin plot with subsequent unpaired *t*-test with Welch correction was selected for analysis to best represent the data distribution. (**D**,**E**) HeLa cells were treated for 4 h with either 5 µM MG132 or DMSO following transfection with UbVIP-i53#3 (**D**) or UbVIP-i53#3 (**E**) and 24 h incubation. The slides were then processed for immunofluorescence and the levels of 53BP1 were compared. Total cell numbers for EV DMSO, EV MG132, #3 DMSO, #3 MG132, #5 DMSO, and #5 MG132, are 148, 125, 60, 65, 85, and 74, respectively.

It has been previously shown that 53BP1 plays an integral part in non-homologous end joining (NHEJ) repair and as such, is present in spontaneous foci formation [29]. Since the introduction of the UbVIPs is hypothesized to reduce the overall level of 53BP1 expression rather than the foci formation, we measured intensity of 53BP1 fluorescence in HeLa cells and focused on the levels of free 53BP1 in the nuclei of transfected cells (Figure 4A). We observed that in the presence of UbVIP-i53 #3 and #5, there was an apparent and consistent reduction in the amount of 53BP1 (Figure 4B). In addition, we noticed that the presence of UbVIPs in the cells (confirmed by staining the cells with an anti-FLAG antibody) significantly reduced the number of foci in the nucleus and decreased the overall amount of 53BP1. Cells from transfected well that do not express UbVIPs were used as a negative control and compared to cells that express UbVIP within the same cell population. Since some variation in 53BP1 expression exists among both the transfected and the untransfected cells, an image analysis software was used to calculate the average level of protein in each group of cells (Figure 4C). Thus, the reduction in the amount of free 53BP1 in the presence of UbVIP-i53 #3 and #5, detected through epifluorescent microscopy further confirms our earlier observations.

Finally, we found that the treatment of proteasome inhibitor MG132, but not DMSO as control, can restore the 53BP1 protein abundance in cells with UbVIP-i53#3 (Figure 4D) and #5 (Figure 4E). Therefore, we concluded that UbVIP-i53 #3 and #5 can lead to proteasome-mediated targeted degradation of 53BP1 by engaging cellular E3 ligases RFWD3 and NEDD4L, respectively.

## 4. Discussion

One critical bottleneck in current PROTACs and other targeted protein degradation technologies is the limitation of E3 ligases being utilized. This is in part due to the difficulty of obtaining specific yet non-inhibitory chemical entity binders for E3 ligases. To solve this problem, structure-based protein design and engineering methods were devised to systematically generate UbV binders for E3 ligases [16,17,31,32]. We therefore reasoned that the utilization of non-inhibitory UbVs as E3 ligase engagers would break down the limitations of PROTACs and other degraders and allow for far greater targeting landscape.

In this work, we provided a proof-of-principle that UbVIP is capable of inducing degradation of an intracellular protein. We proposed several important design features that are crucial for future UbVIP development. First of all, the linker size is a crucial factor for degradation effectiveness, as has previously been shown in PROTACs [33]. Small linkers tend to limit the ability for the PROTACs to bend into the necessary organization to facilitate ubiquitination, while large linkers tend to have too much flexibility, leading to less interactions between the E3 ligase and the target, as well as increasing hook effects as length increases beyond the optimal point [34]. Because of its critical role in positioning of the ligands, the optimal size for a PROTACs linker depends on the structure of the E3 ligase engager and the target protein binder [34]. Here, we included two linker lengths in the design of UbVIP, a medium 8 residue linker and a large 15 residue linker, to get a general idea of the optimal size of the UbVIP linker. For both UbVIP-i53#3 and UbVIP-i53#5, the linker is 8aa, suggesting that medium sized linkers maybe more effective in targeted degradation of 53BP1 using UbVIP. Going forward, further modification to identify optimal linker length may be necessary. However, given the structure similarities between UbVs, 8aa linker is likely also preferred for other UbVIPs. Nevertheless, a comprehensive study of a series of medium sized linkers (e.g., <15aa), as well as some potential linker structural changes (different from the GS linker) would give a more thorough knowledge base of optimized linkers which would likely carry over into future UbVIP molecules.

Secondly, ligand orientations were also compared in this study. In PROTAC structures, orientation has also been found to alter ubiquitination efficiency [35] In terms of UbVIP-i53, the orientation of the E3 engager on the N-terminus was found to be superior in ubiquitinating 53BP1 for several UbVIPs. Therefore, this orientation setup would also likely be optimal among other UbVIPs due to the similarity of the UbV binders; however, the exact structural reason is not known and would be worth investigating in the future.

Thirdly, there is no doubt that the E3 ligase engager module is key for the UbVIP effectiveness. As we showed above, two E3s ligases were capable of degrading 53BP1, while others could not. This is consistent with one of the rationales for the development of UbVIPs, in that the limited number of E3 ligases currently used decreases the capability for PROTACs to target diverse proteins. Here, we tested a total of nine non-inhibitory E3 binding UbV binders for the purpose of developing UbVIP molecules; however, only two UbVs have the capacity to recruit endogenous E3s effectively in cells. While some UbVs have not been fully characterized for their cellular activity, UbVs with validated cellular activity in modulating E3 ligases were found to not be effective in the UbVIP structure. These complications demonstrate both the need and usefulness for similar studies in which UbVs are developed for E3 ligases and tested in UbVIP design. Future generation of more non-inhibitory E3-binding UbVs and thorough studies of more UbVIPs will lead to a large toolkit that could be leveraged by the research community to enable more diverse degradation capacity.

Last but not the least, a thorough proteomics analysis may be required before using the reported UbVIP-i53 molecules in targeted protein degradation experiments. While the specificity of both i53 [29] and UbV^NL.1^ [16] have been validated, UbV^RF.9^ may cross-react with other WD40 domain-containing proteins. Like the off-target effects in drug discovery, whether UbVIP-i53#3 or UbVIP-i53#5 may bind to other proteins to induce 53BP1 degradation will be comprehensively studied in future experiments.

The UbVIP molecules identified here could be modified by other researchers for targeted degradation of other nucleus-localized proteins of interest. For example, proteins involved in transcriptional activation, DNA repair, and cell cycle control can be detrimental in excess, and their control may lead to therapeutic benefits. This study lays the groundwork for a scenario in which UbVIP technology allows for the specific control of such a broad variety of proteins, allowing precise mediation of unhealthy protein concentrations to treat human diseases, including neurological disorders and cancer.

## Figures and Tables

**Table 1:** List of UbVIP constructs and the corresponding identifier number.

Identifier	Construct
UbVIP-i53#1	UbV^RF.5^-8aa-i53
UbVIP-i53#2	UbV^RF.7^-8aa-i53
UbVIP-i53#3	UbV^RF.9^-8aa-i53
UbVIP-i53#4	UbV^3A.2^-8aa-i53
UbVIP-i53#5	UbV^NL.1^-8aa-i53
UbVIP-i53#6	UbV^MDM2^-8aa-i53
UbVIP-i53#7	UbV^RF.5^-15aa-i53
UbVIP-i53#8	UbV^RF.7^-15aa-i53
UbVIP-i53#9	UbV^RF.9^-15aa-i53
UbVIP-i53#10	UbV^3A.2^-15aa-i53
UbVIP-i53#11	UbV^NL.1^-15aa-i53
UbVIP-i53#12	UbV^MDM2^-15aa-i53
UbVIP-i53#13	i53-8aa-UbV^RF.5^
UbVIP-i53#14	i53-8aa-UbV^RF.7^
UbVIP-i53#15	i53-8aa-UbV^RF.9^
UbVIP-i53#16	i53-8aa-UbV^3A.2^
UbVIP-i53#17	i53-8aa-UbV^NL.1^
UbVIP-i53#18	i53-8aa-UbV^MDM2^
UbVIP-i53#19	i53-15aa-UbV^RF.5^
UbVIP-i53#20	i53-15aa-UbV^RF.7^
UbVIP-i53#21	i53-15aa-UbV^RF.9^
UbVIP-i53#22	i53-15aa-UbV^3A.2^
UbVIP-i53#23	i53-15aa-UbV^NL.1^
UbVIP-i53#24	i53-15aa-UbV^MDM2^

i53: 53BP1-binding UbV.

## Data Availability

All data supporting reported results can be found within this manuscript.

## Supplementary Materials

The following supporting information can be downloaded at: https://www.mdpi.com/article/10.3390/biom12040479/s1, Figure S1: Effects of MDM2 and UBE3A UbVs in p53 ubiquitination; Figure S2: Validation of i53 activity in human cell lines; Figure S3: UbVIP-i53 #3 and UbVIP-i53 #5 do not affect natural substrates of the E3 ligases and global ubiquitination; Figure S4: 53BP1 degradation upon expression of UbVIP-i53 #25-#27; Table S1: List of additional UbVIP constructs.
