# Supplementary material for: Targeted Degradation of 53BP1 Using Ubiquitin Variant Induced Proximity
Source: Biomolecules. 2022 Mar 22;12(4):479. doi: 10.3390/biom12040479 (PMC9029692; doi:10.3390/biom12040479)
Supplement: Supplementary file 1 [file biomolecules-12-00479-s001.zip › biomolecules-1610424-supplementary.pdf]

Supplemental Tables and Figures

Table S1. List of additional UbVIP constructs.

Identifier	Construct
UbVIP-i53#25	$UbV^{P2.1}$-8aa-i53
UbVIP-i53#26	$UbV^{P2.3}$-8aa-i53
UbVIP-i53#27	UbV^{XR}-8aa-i53

Figure S1. Effects of MDM2 and UBE3A UbVs in p53 ubiquitination. MDM2 or UBE3A (pre-mixed for 15 min with WT Ub or UbV as indicated) was incubated for 1 h at 37°C with E1, E2, ATP, and Ub (p53 ubiquitination kit from Boston Biochem). Western blots were probed with an anti-p53 antibody to detect poly-ubiquitinated p53. UbVs are not incorporated into chains because their C termini do not contain a di-glycine motif that is required for recognition by the E1 enzyme.

Figure S2. Validation of i53 activity in human cell lines. Two cell lines, HeLa and U2OS, were transfected with i53 expressing vector or with an empty control vector (EV). 24 hours after the transfection the cells were fixed and stained with an antibody against 53BP1. The cells were then observed microscopically to compare the number of foci in EV samples vs i53 expressing samples. The top two images of the micrograph represent HeLa and U2OS cells expressing EV while the bottom two images show the respective cell lines expressing i53 vector.

Figure S3. **UbVIP-i53#3 and UbVIP-i53#5 do not affect natural substrates of the E3 ligases and global ubiquitination.** HEK 293T cells were transfected with the UbVIP-i53 constructs and cells lysed after 24 h and whole cell lysates were subjected to SDS-PAGE and immunoblotting using antibodies against (**A**) RPA32, an RFWD3 substrate and (**B**) RhoB, a substrate of NEDD4L and compared with cells transfected with empty vector. Presence of UbVIP was confirmed by blotting for FLAG. β-Actin was used as a loading control and anti-ubiquitinated antibody (clone FK2) was used to detect global protein ubiquitination. (**C**) Cell survival following the transfection of UbVIP. Cell viability for both HEK 293 and HeLa cells following UbVIP expression was determined using the MTT assay and the absorbance data was processed in Prism software using paired Student T test. The absorbance data was converted to percent survival, with empty vector transfectants established as 100%.

Figure S4. 53BP1 degradation upon expression of UbVIP-i53 #25-#27. (**A**) Pipeline for determining expression of UbVIP in mammalian cells. HEK293 cells were transfected with the UbVIP-i53 constructs and cells lysed after 24h and whole cell lysates were subjected to SDS-

PAGE and immunoblotting using an antibody against the FLAG tag and 53BP1. (**B**) Western blot analysis of HEK293 cells transfected with UbVIP-i53#25, #26, or #27 compared to untransfected control. The identifier numbers are as described in **Table S1**. 53BP1 protein abundance was measured using ImageJ software and normalized to β-Actin. Values indicated are relative to the untransfected control.

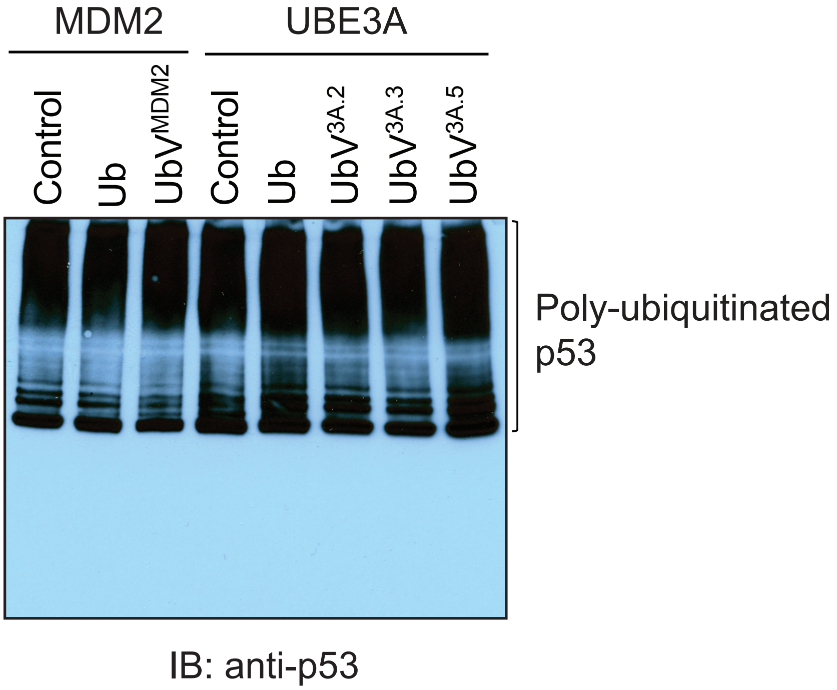

Figure S1. **Effects of MDM2 and UBE3A UbVs in p53 ubiquitination.** MDM2 or UBE3A (premixed for 15 min with WT Ub or UbV as indicated) was incubated for 1 h at 37°C with E1, E2, ATP, and Ub (p53 ubiquitination kit from Boston Biochem). Western blots were probed with an anti-p53 antibody to detect poly-ubiquitinated p53. UbVs are not incorporated into chains because their C termini do not contain a di-glycine motif that is required for recognition by the E1 enzyme.

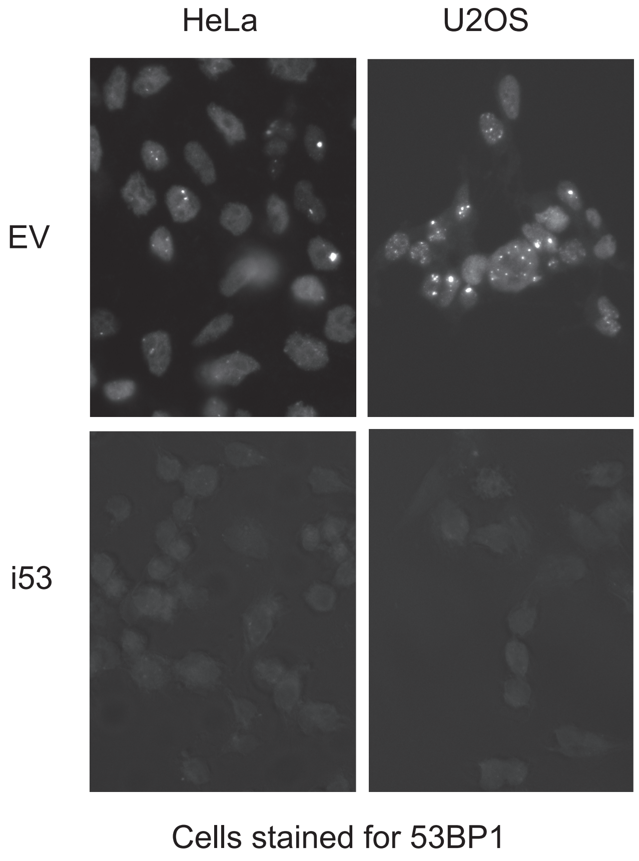

Figure S2. Validation of i53 activity in human cell lines. Two cell lines, HeLa and U2OS, were transfected with i53 expressing vector or with an empty control vector (EV). 24 hours after the transfection the cells were fixed and stained with an antibody against 53BP1. The cells were then observed microscopically to compare the number of foci in EV samples vs i53 expressing samples. The top two images of the micrograph represent HeLa and U2OS cells expressing EV while the bottom two images show the respective cell lines expressing i53 vector.

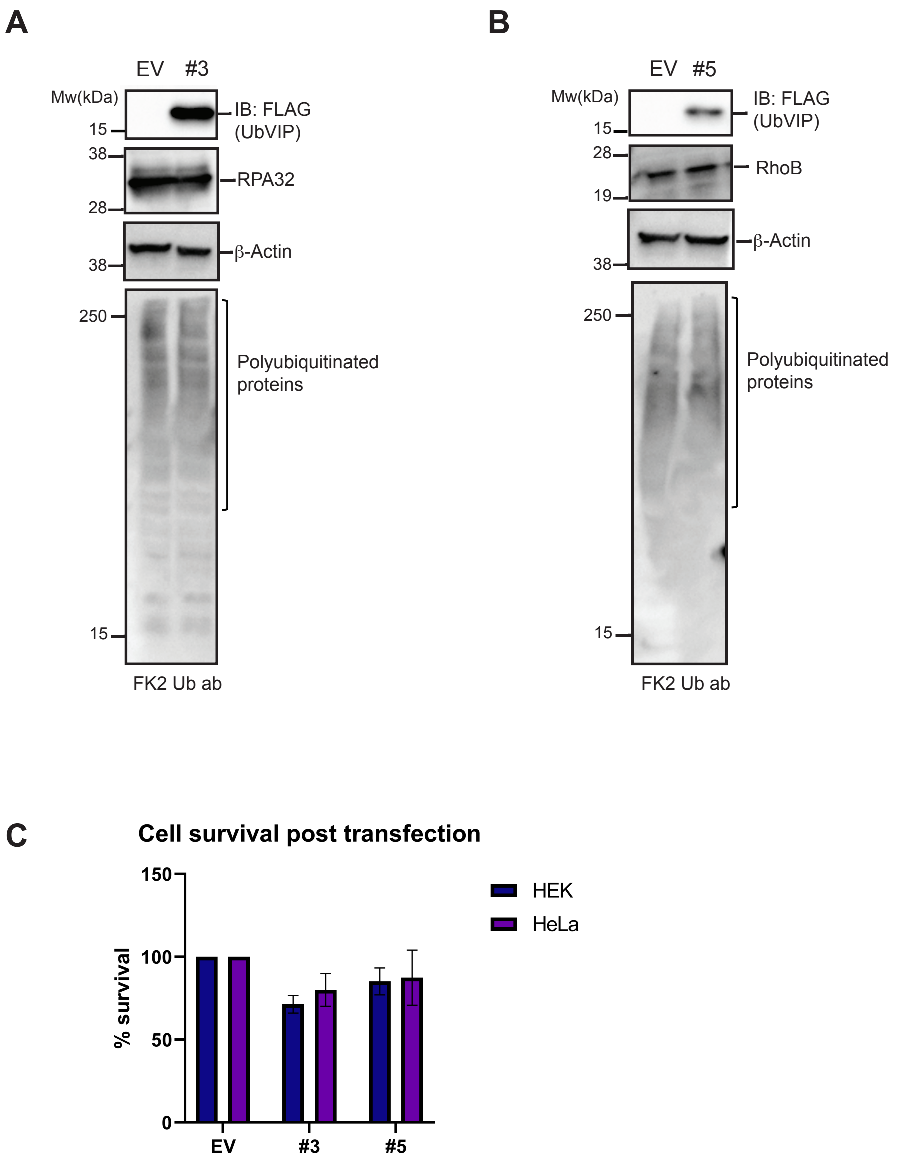

Figure S3. UbVIP-i53#3 and UbVIP-i53#5 do not affect natural substrates of the E3 ligases and global ubiquitination. HEK 293T cells were transfected with the UbVIP-i53 constructs and cells lysed after 24 h and whole cell lysates were subjected to SDS-PAGE and immunoblotting using antibodies against (**A**) RPA32, an RFWD3 substrate and (**B**) RhoB, a substrate of NEDD4L and compared with cells transfected with empty vector. Presence of UbVIP was confirmed by blotting for FLAG. β-Actin was used as a loading control and anti-ubiquitinated antibody (clone FK2) was used to detect global protein ubiquitination. (**C**) Cell survival following the transfection of UbVIP. Cell viability for both HEK 293 and HeLa cells following UbVIP expression was determined using the MTT assay and the absorbance data was processed in Prism software using paired Student T test. The absorbance data was converted to percent survival, with empty vector transfectants established as 100%.

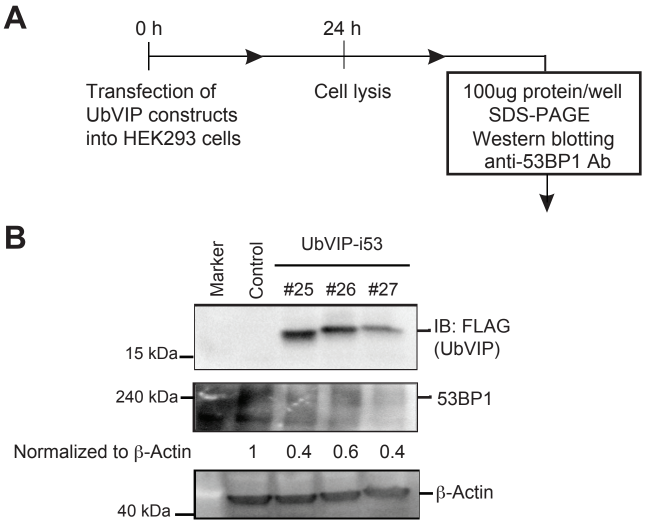

Figure S4. 53BP1 degradation upon expression of UbVIP-i53 #25-#27. (**A**) Pipeline for determining expression of UbVIP in mammalian cells. HEK293 cells were transfected with the UbVIP-i53 constructs and cells lysed after 24h and whole cell lysates were subjected to SDS-PAGE and immunoblotting using an antibody against the FLAG tag and 53BP1. (**B**) Western blot analysis of HEK293 cells transfected with UbVIP-i53#25, #26, or #27 compared to untransfected control. The identifier numbers are as described in **Table S1.** 53BP1 protein abundance was measured using ImageJ software and normalized to b-Actin. Values indicated are relative to the untransfected control.
